# Biased MAIT TCR Usage Poised for Limited Antigen Diversity?

**DOI:** 10.3389/fimmu.2020.01845

**Published:** 2020-08-18

**Authors:** Michael N. T. Souter, Sidonia B. G. Eckle

**Affiliations:** Department of Microbiology and Immunology, The Peter Doherty Institute for Infection and Immunity, The University of Melbourne, Melbourne, VIC, Australia

**Keywords:** MR1, MAIT cells, antigen diversity, TCR repertoire diversity, T cell subsets

## Abstract

Mucosal-associated invariant T (MAIT) cells are a subset of unconventional T cells that recognize the evolutionarily conserved major histocompatibility complex (MHC) class I-like antigen-presenting molecule known as MHC class I related protein 1 (MR1). Since their rise from obscurity in the early 1990s, the study of MAIT cells has grown substantially, accelerating our fundamental understanding of these cells and their possible roles in immunity. In the context of recent advances, we review here the relationship between MR1, antigen, and TCR usage among MAIT and other MR1-reactive T cells and provide a speculative discussion.

## Introduction

Conventional T cells recognize peptide antigens presented by the major histocompatibility complex (MHC) class I or class II (MHC-I or MHC-II) molecules and elicit a cellular immune response to provide anti-microbial and anti-tumoral immunity for the host. Conventional T cells utilize a cell surface bound T cell receptor (TCR) to recognize peptide-MHC complexes presented on the surface of antigen-presenting cells (APCs). The TCR is a heterodimer comprised of a TCR α- and β-chain, each consisting of a variable (V) and constant (C) domain. Three sets of finger-like extensions on the TCRα- and β-chain variable domains, known as complementarity determining loops (CDR1, CDR2, and CDR3), are responsible for recognizing the peptide-MHC-complex and are collectively unique to each TCR. CDR1 and CDR2 loops are germline-encoded by the V gene segments, while the CDR3 loops are formed from somatic recombination of the V gene segments with a joining (J) (V-J) and/or diversity (D) gene segment (V-D-J) during T cell development (1). Together with random nucleotide additions and deletions in the CDR3 loop gene regions, this creates an enormous diversity of TCRs, clonally distributed amongst T cells (2).

**Figure 1:**
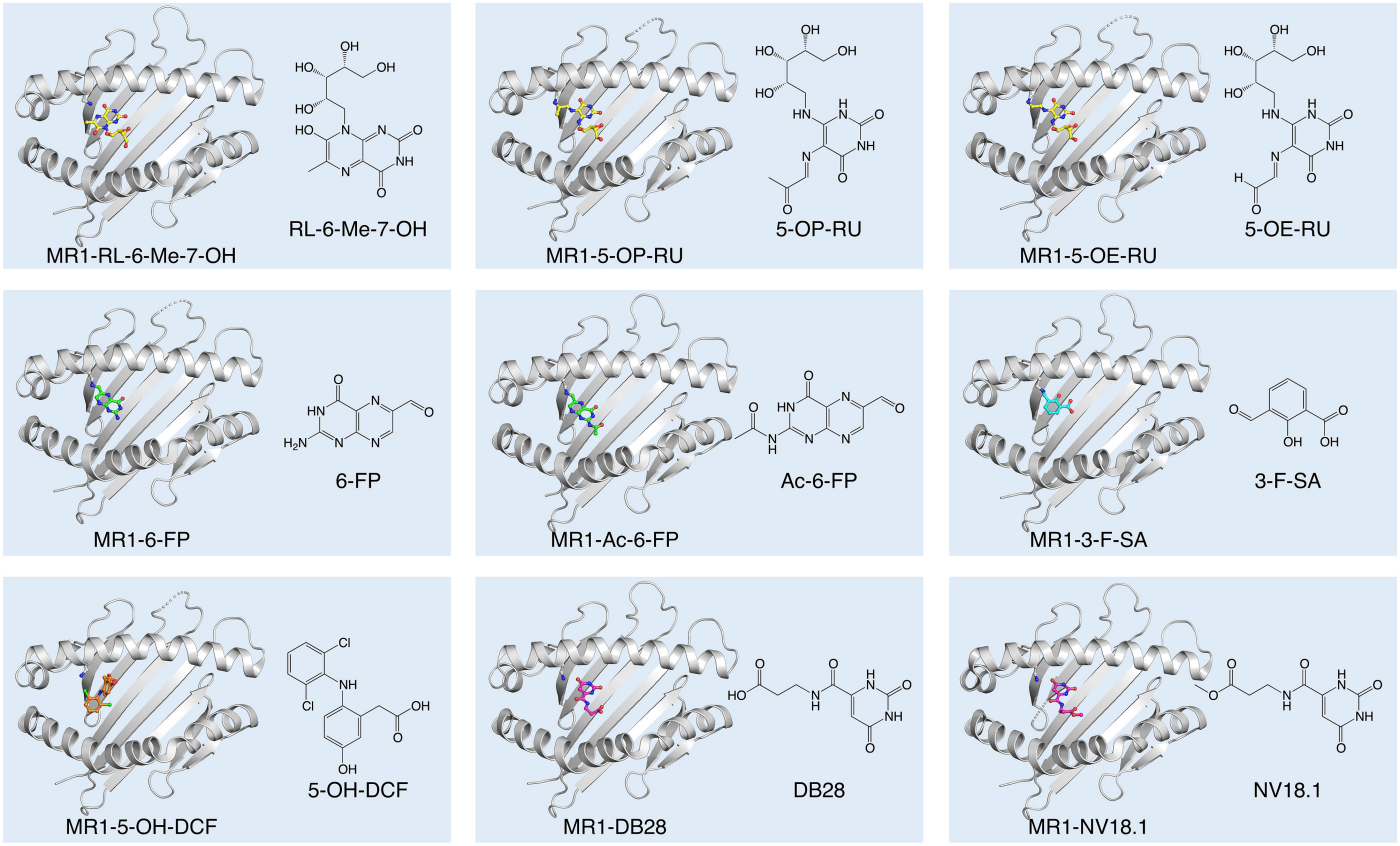
Diversity of small molecule ligands presented by MR1. Cartoon display (light gray) of the MR1 antigen-binding cleft (top-view) and ball-and-stick display of the antigen (colored) based on the protein data bank (PDB) deposited crystal structures, featuring the human A-F7 MAIT TCR in complex with human MR1-RL-6-Me-7-OH [PDB ID: 4L4V (27)], MR1-5-OP-RU and MR1-5-OE-RU [PDB IDs: 4NQC, 4NQE (26)], MR1-6-FP [PDB ID: 4L4T (27)], MR1-Ac-6-FP [PDB ID: 4PJF (28)], MR1-3-F-SA and MR1-5-OH-DCF [PDB IDs: 5U6Q, 5U72 (29)], and MR1-DB28 and MR1-NV18.1 [PDB IDs:6PVC and 6PVD (30)].

Mucosal-associated invariant T (MAIT) cells are a subset of unconventional T cells that are restricted by a monomorphic MHC-I-like molecule, known as MHC class I related protein 1 (MR1) (3–6). In contrast to conventional T cells, MAIT cells express a “semi-invariant” αβ TCR, meaning they typically express the same TCRα chain paired to a preferred array of TCRβ chains (3). In humans, the “classical MAIT TCR” comprises a TCRα chain encoded by the V gene segment *TRAV1-2* juxtaposed to the J gene segment *TRAJ33, TRAJ20*, or *TRAJ12* (5, 7–9) that pairs preferentially with a TCRβ chain encoded by *TRBV6* or *TRBV20 V* gene segment family members (7). In mice, the MAIT TCR is composed of homologous gene segments, a *TRAV1-TRAJ33* TCRα chain, that pairs with a TCRβ chain composed of *TRBV19* or *TRBV13 V* gene segments, both of which are murine orthologous segments of human *TRBV6* (3, 5, 10). MAIT cells are abundant in healthy adults, representing on average 3% of blood T cells (11) and can be found throughout peripheral tissues (6, 7, 10, 12–23). The frequency of MAIT cells in laboratory mice is distinctly lower than in humans, although murine MAIT cells are also found in many peripheral organs (24, 25). The prototypical antigen presented by MR1 to MAIT cells is the small molecule 5-(2-oxopropylideneamino)-6-D-ribitylaminouracil (5-OP-RU), an adduct of the riboflavin biosynthetic precursor 5-amino-6-D-ribitylaminouracil (5-A-RU) and methylglyoxal (26) (Figure 1). See recent reviews for details on the riboflavin biosynthesis and formation of 5-OP-RU from 5-A-RU (31, 32). Riboflavin biosynthesis is absent in mammals. Thus, by recognizing 5-OP-RU (25, 33, 34), and potentially other riboflavin-based ligands presented by MR1 (35), MAIT cells are able to sense a broad range of riboflavin biosynthesis proficient microbes in a highly conserved, innate-like manner, reviewed in (32). Human MAIT cells stimulated with 5-OP-RU rapidly secrete T helper (Th)1 and Th17 type cytokines (11, 36, 37) as well as cytotoxic granules (38). In mice, lung infection with riboflavin-synthesizing bacteria or co-administration of synthetic 5-OP-RU with adjuvant leads to a significant expansion of MAIT cells with Th1/17 cytokine secreting capacity (25, 34, 39), enabling MAIT cells to contribute to protection against several pathogens, including *Klebsiella pneumoniae* (40), *Mycobacterium bovis* BCG (41), *Francisella tularensis* (39), *E. coli* (42), *Legionella longbeachae* (34), and *Clostridium difficile* (43). Thus, observations to date suggest MAIT cells are poised, but perhaps not limited to, protecting peripheral tissues from microbial pathogen or commensal breach. In particular, MAIT cells have recently been shown to contribute to tissue repair at barrier sites (44–47). MAIT cells may also be involved in the tumoral immune response (48–52), however, elevated MAIT cell numbers at the tumor site in some cancers correlate with a poorer prognosis (49, 52). Notably, MAIT cells appear to be subject to a similar fate as conventional T cells during the anti-tumoral immune response, namely: T cell exhaustion, altered functional response, altered frequency, and drug sensitivity (50, 52–57). A cytokine-modulated (IL-7, IL-12, IL-18) tumor response that occurs independent of, or concurrent with, TCR stimulation should also be considered in the context of tumoral immunity, as MAIT cells are known to respond to inflammatory stimuli in this manner (15, 58, 59). Furthermore, MAIT cells from healthy donors can efficiently lyse MR1 proficient tumor cells presenting microbial agonists such as 5-OP-RU, suggested as a potential strategy to harness the MAIT cell response therapeutically (56). Perhaps similar in mechanism, disruption of barrier tissues (i.e., colorectal cancers) by tumors may allow invasive growth of commensal bacteria, providing a source of microbial ligand in the context of an inflammatory environment which may trigger anti-tumor MAIT cell responses (48–50, 60). Much is still unknown regarding the response by MAIT cells in the tumoral environment, particularly whether tumor associated, MAIT cell specific MR1 ligands exist and the factors that might drive MAIT cell to become pro- or anti-tumoral. MAIT cells have, however, attracted some interest as a potential immunotherapeutic target as they possess a number of favorable attributes such as a high precursor frequency, wide tissue distribution, potent cytokine response and cytotoxicity and a donor unrestricted nature (61).

## The Riboflavin-Based MR1 Ligands

Independent observations from Gold et al. and Bourhis et al. demonstrated that a wide range of bacteria and yeasts, and their supernatants, are capable of stimulating MAIT cells in an MR1-dependent manner (36, 62). On the assumption that MR1 would likely adopt a “MHC-I-fold” (63) in the presence of ligand, Kjer-Nielsen et al. folded soluble recombinant MR1 proteins in the presence of bacterial supernatant to capture ligands in the form of stable MR1-ligand-complexes (35). This approach of ligand-capture, combined with mass-spectrometry, and subsequent genetic manipulation of the riboflavin biosynthetic pathway in bacteria, led to the discovery of the pyrimidines; 5-OP-RU and 5-(2-oxoethylideneamino)-6-D-ribitylaminouracil (5-OE-RU), and the substantially less potent, cyclised ribityllumazines; 7-hydroxy-6-methyl-8-D-ribityllumazine (RL-6-Me-7-OH); and 7-dimethyl-8-D-ribityllumazine (RL-6,7-diMe) as riboflavin-based, MR1-presented, MAIT cell stimulating antigens (26, 27, 32, 35) (Figure 1). While both 5-OP-RU and 5-OE-RU were detected in the supernatant of *Escherichia coli* and *Salmonella enterica* serovar Typhimurium, 5-OP-RU is the dominant MAIT cell antigen found in these bacterial supernatants (26). Nevertheless, MR1 tetramers loaded with 5-OE-RU stain a similar proportion of T cells compared to those loaded with 5-OP-RU (26) and synthetic versions of both antigens are similarly potent (64, 65). 5-A-RU co-incubated with methylglyoxal is, however, more potent in activating MAIT cells than 5-A-RU co-incubated with glyoxal (26, 66), presumably due to differences in the catabolic and metabolic kinetics of 5-OP-RU and 5-OE-RU (64). 5-OP-RU has emerged as the preferred antigen for research, both as synthetic antigen and loaded in MR1 tetramers. Accordingly, MR1-5-OP-RU tetramers have become the gold standard for identifying MAIT cells (67), enabling more accurate MAIT cell detection than using antibodies specific for surrogate markers (TRAV1-2, CD161, and CD8) (11). MR1 presentation of 5-OP-RU and 5-OE-RU involves the formation of a Schiff base between the Lys43 of MR1 and a residual carbonyl group on the ligand. This Schiff base, unique to MR1 ligand presentation amongst MHC molecules, stabilizes, and guides 5-OP-RU and 5-OE-RU into their position on MR1 in the A'-pocket amongst a cradle of aromatic residues (26). Notably, even in the absence of the Schiff base, by mutating Lys43 in MR1 to Ala (68), 5-OP-RU is presented by MR1 in the identical position (26), although the stability of this complex is markedly reduced (28). Unlike the pyrimidine ligands, RL-6-Me-7-OH does not form a Schiff base with MR1, however residues within the A'-pocket of MR1 help orient the ligand into a similar position (27). In a physiological context, neutralization of MR1 Lys43 by covalently bound ligand is essential for complete MR1 folding and trafficking from the endoplasmic reticulum (ER) to the cell surface (69).

**Figure 2:**
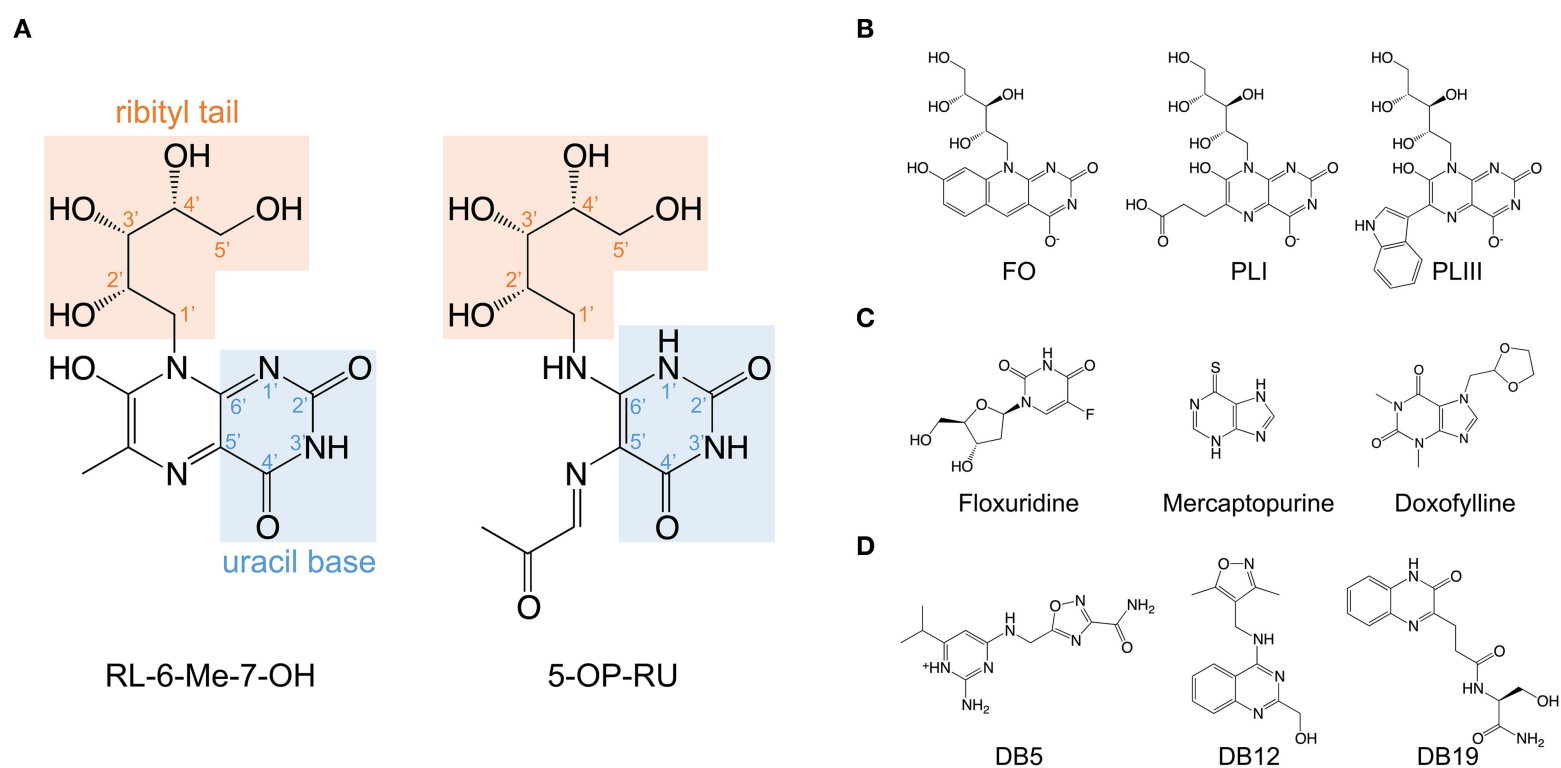
Structures of the riboflavin-based and related MR1 ligands. **(A)** Chemical structure of 7-hydroxy-6-methyl-8-D-ribityllumazine (RL-6-Me-7-OH) and 5-(2-oxopropylideneamino)-6-D-ribitylaminouracil (5-OP-RU) annotated with the position numbers for relevant functional groups. **(B)** MR1 ligands isolated from soluble recombinant MR1 expressed with *E. coli* or *M. smegmatis*; lumazines 6-(2-carboxyethyl)-7-hydroxy-8-ribityllumazine (photolumazine I; PLI) and 6-(1H-indol-3-yl)-7-hydroxy-8-ribityllumazine (photolumazine III; PLIII) and the riboflavin analog 7,8-didemethyl-8-hydroxy-5-deazariboflavin (FO). **(C,D)**. A selection of MAIT cell agonist and non-agonist MR1 ligands identified from *in silico* screens of multiple chemical libraries; mercaptopurine, floxuridine, doxofylline, and 2-amino-4-(((3-carbamoyl-1,2,4-oxadiazol-5-l)methyl)amino)-6-isopropylpyrimidin-1-ium (DB5), (4-(((3,5-dimethylisoxazol-4-yl)methyl)amino)quinazolin-2-yl)methanol (DB12) and (S)-N-(1-amino-3-hydroxy-1-oxopropan-2-yl)-3-(3-oxo-3,4-dihydroquinoxalin-2-yl)propenamide (DB19).

All classical MAIT TCRs studied to date recognize MR1 presenting riboflavin-based antigens using a surprisingly consistent mode of docking, orthogonally over the cleft of MR1 and forming multiple contacts with aromatic residues that surround the ligand-binding site, notably residues Tyr62 and Tyr152 (26–28, 68, 70). The TCR also directly contacts the antigen, which occupies only 0.6% of the exposed surface in the MR1 cleft (70). Crucial to antigen recognition by classical MAIT TCRs is Tyr95α, a highly conserved residue in the CDR3α loop which directly contacts the 2′-OH group on the antigen ribityl moiety (Figure 2A) (26–28, 68). Structure-function studies with synthetic derivatives of 5-OP-RU and other riboflavin-based antigens have provided valuable insights on which chemical group modifications affect ligand antigenicity within the MAIT-MR1 axis (64, 65). While improving ligand stability, modification of the pyrimidine backbone of 5-OP-RU (C-5 and C-6) (Figure 2A), had only small effects on altering antigen potency (64), perhaps unsurprising as these atoms make no direct contact with the MAIT TCR (26, 28, 64, 65). In contrast, modifications to the ribityl moiety of 5-OP-RU and ribityllumazine ligands had profoundly negative effects on MAIT TCR recognition and antigen potency (65, 71). Particularly, removal of the ribityl 2′- and 3′-OH groups or the entire ribityl tail largely abolished MAIT TCR recognition of relevant 5-OP-RU analogs (65, 71). In contrast, shortening of the ribityl tail, while preserving the 2′- and 3′-OH groups (ethyl- or propyl-5-OP-RU analogs) did not appreciably affect MAIT TCR recognition (65, 71). Notably however, 2′-OH was not required for MAIT TCR recognition of a modified analog of the ribityllumazine RL-6-Me-7-OH (72). Structural modeling suggested the modified ribityl tail of the 2′-deoxy-RL-6-Me-7-OH is flexible enough to be oriented for recognition by the MAIT TCR Tyr95α via a hydrogen bond with the 5′-OH in place of the 2′-OH group (Figure 2A) (72). Although MAIT cells could recognize MR1 presenting the 2-deoxyribityllumazine, this interaction was not sufficient to lead to MAIT cell activation (72), stressing that distinct requirements exist for MAIT cell activation that are not satisfied by TCR engagement alone. Thus, the series of altered metabolite ligands helped to define the boundaries of riboflavin-based antigen diversity tolerated by the MAIT TCR and emphasized the importance of the ribityl tail and Tyr95α in MAIT TCR recognition. In light of this, new microbial ligands have been identified that share the ribityl moiety and some structural features with the riboflavin-based antigens (73). The lumazines 6-(2-carboxyethyl)-7-hydroxy-8-ribityllumazine (photolumazine I; PLI) and 6-(1H-indol-3-yl)-7-hydroxy-8-ribityllumazine (photolumazine III; PLIII) and the riboflavin analog 7,8-didemethyl-8-hydroxy-5-deazariboflavin (FO) were isolated from soluble recombinant MR1 expressed in the context of live bacterial infection (Figure 2B) (73). Although all three ligands had ribityl moieties, only PLI and PLIII, possible adducts of 5-A-RU, were antigenic to MAIT cells, while FO was antagonistic (73). Similarly, three pyrimidines, mercaptopurine, floxuridine, and doxofylline (Figure 2C) were identified as putative MR1 ligands in an *in silico* screen of a range of small organic molecules, including drugs, drug metabolites, and drug-like molecules (29). In cellular assays, floxuridine and mercaptopurine were weakly agonistic but did not upregulate MR1 to detectable levels, while doxofylline weakly upregulated MR1 but was not agonistic (29). Further *in silico* screening of commercial compound libraries recently identified seven weakly agonistic MR1 ligands (Figure 2D) (30). None of the novel agonist ligands were as potent as 5-OP-RU in cellular assays or were able to upregulate MR1 surface expression, likely due to the inability to form a Schiff base with MR1 Lys43 (30). In contrast, in the same study, two pyrimidines were identified; 3-[(2,6-dioxo-1,2,3,6-tetrahydropyrimidin-4-yl)formamido] propanoic acid (DB28) and its derivative methyl 3-[(2,6-dioxo-1,2,3,6-tetrahydropyrimidin-4-yl)formamido] propanoate (NV18.1) that significantly modulated MR1 surface expression (30). These ligands bound to MR1 non-covalently (Figure 1), causing MR1 to be retained in the ER and prevented surface expression (30). Importantly, unlike the other ligands, DB28 and NV18.1 were non-stimulatory to PBMC-derived TRAV1-2^+^ MAIT cells (30).

## Characteristics of the MAIT TCR Repertoire

The classical MAIT TCRα rearrangements (*TRAV1-2-TRAJ33/20/12*) are highly conserved between genetically unrelated individuals, also referred to as public TCRα chain usage (3, 5). Collectively, these TCRα rearrangements account for the majority (~95%) of MAIT TCR clonotypes in human blood (7–9, 11, 62, 74). Nevertheless, variations of the classical MAIT TCR CDR3α region exist, typically at one or two non-germline-encoded residue positions (CAXXDSSYKLIF, CAVXXXDYKLSF, and CAXXDSNYQLIW for TRAJ12, TRAJ20, and TRAJ33 MAIT CDR3α rearrangements, respectively) (5, 11, 74). However, MR1-reactive clones responding to different microbes (bacteria and yeast) are characterized by more extensive non-germline-encoded differences within the TCRα chain, including what appeared to be microbe-specific MR1-reactive clonotypes in some instances (8). Similarly, systemic infection of healthy volunteers with *S. enterica* serovar Paratyphi A transiently altered the MAIT TCR repertoire within individuals at the clonotypic level (75). In this case, circulating, overrepresented MAIT clonotypes were reduced during infection but restored to homeostatic proportions at the resolution of infection (75). TCRs from MAIT clonotypes that expanded during infection showed greater stimulatory potential than TCRs from contracted MAIT clonotypes in response to synthetic riboflavin-based antigens or bacterial supernatant (*S. enterica* Paratyphi A or *E. coli*) (75), suggesting TCR-dependent but not microbe-specific differences in MAIT clonotypic responses. In mice, stimulation of circulating MAIT cells with the different riboflavin-based antigens induced activation of separate cell clusters based on their activated surface phenotype (66), perhaps involving preferential stimulation of clonal MAIT TCRs. Similarly, in mice, preferential accumulation of MAIT cells with divergent functional phenotypes occurs in response to acute infection by different bacterial species (*S. enterica* serovar Typhimurium or *Legionella longbeachae*) (34, 46, 76), however, divergence in the functional response of MAIT cells has not been correlated with TCR usage in mice. Interestingly, the TCRα chain *TRAV1-2-TRAJ33* rearrangement is also found in some CD161^−^TRAV1-2^+^ T cells, and nearly one third of those *TRAV1-2-TRAJ33* rearranged TCRs express Tyr95α (74). Whether these cells are also MR1-reactive is not known, although it should be noted that other MHC-reactive T cells and a subset of CD1b-restricted T cells also utilize the *TRAV1-2* gene segment (77–79). A minority of MAIT TCRα *TRAV1-2* rearrangements occur with non-classical junctional regions (non-*TRAJ12/20/33*), some of which do not contain the Tyr95α residue yet still confer MR1-5-OP-RU reactivity (11). These observations indicate that other factors must account for recognition of 5-OP-RU among “MAIT TCR outliers,” as in one case determined by crystallographic analysis (80).

Among MAIT cells, TCRβ chain composition provides the greatest source of variability within the TCR repertoire (7, 8, 10, 11, 28, 74). Despite this, *TRBV6* is a dominant *TRBV* gene segment used amongst MAIT TCRs, although some variations exist and are dependent on the TCRα junctional segment (7, 74). For instance, *TRAV1-2-TRAJ12* TCRα chains more commonly pair with *TRBV6* (>80%), compared to *TRAV1-2-TRAJ33* and *TRAV1-2-TRAJ20* rearranged TCRα chains which pair roughly evenly with TCRβ chains encoded by *TRBV6* and *TRBV20* family members (7, 74). Up to one quarter of paired MAIT TCRs express TCRβ chains that are not from *TRBV6* or *TRBV20* V segment family members (7, 74). In line with the less stringent nature of TCRβ chain usage, the CDR3β region among classical MAIT TCRs are non-germline-encoded and quite hypervariable, ranging from 9 to 19 amino acids in length and containing no discernible sequence motifs (9, 28, 74). A comparison of seven canonical MAIT TCRs (*TRAV1-2-TRAJ33* encoded TCRα chain paired with a *TRBV6-1* encoded TCRβ chain) with similar CDR3α loops and highly variable CDR3β loop length and composition revealed how CDR3β hypervariability impacts on MAIT TCR recognition of MR1 (28). All of the TCRs bound to MR1-5-OP-RU within a similar range of affinities (K_deq_ ~2 μM), with the exception of one TCR that bound MR1-5-OP-RU weaker (K_deq_ ~9.1 μM) (28). Structural analysis of four of the TCRs bound to MR1-5-OP-RU, including the weaker affinity TCR, demonstrated that the overall TCR footprint onto MR1 was conserved (28). Not surprisingly, the greatest variability in MR1 engagement was from contacts by the TCRβ CDR loops and framework regions that differed between TCRs; yet an overall similar buried surface area (BSA) at the interface was achieved (28). CDR3β loop sequence impacted on conformational flexibility of this loop as well as MR1 residues contacted. Importantly, in some structures the CDR3β loop made direct contact with 5-OP-RU, thus fine-tuning MR1 recognition in an antigen-dependent manner (28).

The MAIT TCR repertoire varies throughout the course of life (fetal, neonate, young, and old) and has now been examined in some detail, revealing consistent changes in MAIT cell frequency as we age (7, 9, 74, 80, 81). The overall frequency of MAIT cells in blood increases rapidly from low numbers early in life (11, 82), typically over the first two-to-three decades, declining slowly thereafter (83, 84). In addition, the diversity in clonotypes decreases as certain MAIT cell clones expand over time (84). This is most pronounced in older adults (>65 years), where a small number of private (non-shared) MAIT cell clonotypes dominate the MAIT cell pool (84). It is likely that expansion of naïve MAIT cells in young individuals is a consequence of microbial exposure following thymic egress (36, 45, 82, 85, 86). Interestingly, a large proportion of CD161^+^ T cells found in cord blood express the canonical MAIT TCRα chain (TRAV1-2-TRAJ33) but do not recognize MR1-5-OP-RU tetramers (81). These non-reactive cells are not readily detectable in adults (11, 84), suggesting they do not expand akin to MR1-5-OP-RU-reactive MAIT cells. In this regard, some of these cells might feature a TCRβ chain which prohibits reactivity to MR1 presenting riboflavin-based antigens in favor of other antigens furnished by MR1, some of which may be less abundant or less stimulatory than the riboflavin-based antigens for MAIT cells expressing classical MAIT TCRs (28, 35, 80) or may trigger peripheral tolerance to avoid autoreactive or allergic responses (87). Accordingly, a pairwise comparison of MAIT TCR sequences in cord blood revealed much broader TCRβ diversity compared to those from MAIT cells from adult blood, further highlighting the effect of peripheral exposure on the narrowing of the MAIT TCR repertoire (84).

## Non-Riboflavin Based MR1 Ligands

In addition to the well-defined riboflavin (vitamin B2)-based MR1-ligands which are classified as pyrimidines (5-OP-RU and 5-OE-RU) and ribityllumazines (RL-6-Me-7-OH and RL-6,7-diMe), MR1 also presents other classes of small molecules. They include a range of pterins, namely the photodegradation product of folic acid (vitamin B9), known as 6-formylpterin (6-FP) (35), the synthetically acetylated form of 6-FP, acetyl-6-formylpterin (Ac-6-FP) (28), the photodegradation product 2,4-diamino-6-formylpteridine (2,4-DA-6-FP) of the drugs aminopterin and methotrexate which are synthetic derivatives of folic acid (29), and a synthetic derivative of Ac-6-FP, 2-acetylamino-4-hydroxy-6-formylpteridine dimethyl acetal (33). See recent review for details on the photodegradation of folic acid (32). 6-FP, Ac-6-FP, and 2,4-DA-6-FP form a Schiff base with MR1 Lys43 based on crystal structures (28, 29, 35). Whilst the relevant formyl group of the synthetic derivative of Ac-6-FP, 2-acetylamino-4-hydroxy-6-formylpteridine dimethyl acetal, is blocked (33), partial hydrolysis of the acetal might generate an aldehyde capable of reacting with Lys43 of MR1. Indeed the latter, just like all other listed pterins, caused cell surface upregulation of MR1 (26, 28, 29, 33, 35). In contrast, an equivalently modified version of 6-FP, 2-amino-4-hydroxy-6-formylpteridine dimethyl acetale, did not cause MR1 surface upregulation (33). In peripheral blood of some healthy donors, rare subsets of MR1-Ac-6-FP and MR1-6-FP reactive T cells have been described based on tetramer staining (80), as detailed in the next section. Also, some canonical MAIT TCRs form notable interactions with MR1-6-FP (27) and Ac-6-FP (28), involving direct molecular contact between TCR and Ac-6-FP (28) but not 6-FP (27). However, 6-FP and Ac-6-FP typically do not stimulate MAIT cells (28, 35). Instead 6-FP and Ac-6-FP are mostly known as competitive inhibitors of activation by riboflavin-based antigens such as 5-OP-RU (27, 28). Also 2-acetylamino-4-hydroxy-6-formylpteridine dimethyl acetal acts as a potent competitive inhibitor (33). So far Ac-6-FP remains the most potent competitive inhibitor of MAIT cell activation by 5-OP-RU and other pyrimidine antigens, both *in vitro* in human and mouse cell line assays and *in vivo* in mice (29). The acetyl group in Ac-6-FP which is absent in 6-FP forms additional van der Waals interactions with MR1 as well as a hydrogen bond, correlating with an 8°C increase in the stability of MR1-Ac-6-FP in comparison to MR1-6-FP (28). This, as well as other factors, such as solubility, molecule stability, and capacity for cellular uptake likely contribute to the potency of Ac-6-FP as a competitive inhibitor.

The same small molecule drug screen that identified three pyrimidines as potential MR1 ligands (described above) also identified 19 non-pyrimidine MR1 ligands, which belonged to one or more of the following diverse classes of molecules: aromatic aldehyde, aromatic carboxylate, phenol alinine, flavone, isoflavone, enone, quinone (29). Most of these non-pyrimidine ligands could not stimulate a MAIT TCR reporter cell line, and in crystal structures analyzed, no TCR contacts with the MR1 ligands were observed. All of the non-stimulatory ligands tested, including the salicylic acid analog 3-formylsalicylic acid (3-F-SA) (Figure 1), competitively inhibited MAIT cell activation to a similar or lesser extent as 6-FP. Some non-pyrimidine ligands were able to stimulate a MAIT TCR reporter cell line to varying degrees, of which diclofenac was most potent and whose activity was assigned to one of its metabolites, 5-OH-diclofenac (29) (Figure 1). Unlike the pyrimidine and ribityllumazine antigens which are contacted in a hydrogen bond by a conserved residue in the MAIT TCRα chain (Tyr95α), the 5-OH group of 5-OH-diclofenac was contacted in a hydrogen bond by Glu99 from the CDR3β loop. This matched the observation that 5-OH-diclofenac only activated one of a panel of MAIT TCR reporter cell lines, indicating that the MAIT TCRβ chain was able to “fine-tune” responsiveness to certain ligands (29), in line with an emerging concept that some MAIT cells are capable of discriminating between different classes of antigens based on differences in the MAIT TCRβ chain (28, 73, 80, 88, 89). A weak stimulator, 5-formyl-salicylic acid, also only activated one of a panel of MAIT TCR reporter cell lines, suggestive of a similar mechanism (29). Except for diclofenac/5-OH-DCF, all MR1 ligands, of which crystal structure bound to MR1 and complexed with a MAIT TCR were determined as part of this study, featured a Schiff base with Lys43 of MR1 (26, 27, 29) (Figure 1).

In summary, accumulated data thus far (26–29, 35) indicate that MR1 has the capacity to bind structurally diverse small molecules (150–400 Da). Most of the MR1 ligands (except for RL-6-Me-7-OH and Diclofenac/5-OH-diclofenac) form a Schiff base with Lys43 of MR1. However, regardless of the Schiff base, all ligands are located broadly within a similar location in the A'-pocket of MR1, although some are displaced; or oriented differently, involving mostly rotations with broadly similar depositions of the planes of the aromatic rings (26, 27, 29, 35). One notable exception is diclofenac/5-OH diclofenac, whose ring was not deposed in the same plane, rather, the central ring was essentially perpendicular to that of any of the other MR1 ligands (Figure 1) (29). Both in the context of bacterial infections (73, 88), cancer and steady-state (44, 90) there is evidence for the existence of additional MR1 ligands, including some that do not appear to be pyrimidines or ribityllumazines (73, 88), although the chemical identities of these remain to be defined. Some of these ligands appear to be recognized by T cells that do not necessarily share the phenotype of MR1-5-OP-RU specific MAIT cells, described in detail in the next section.

## Diverse MR1-Reactive T Cells

**Figure 3:**
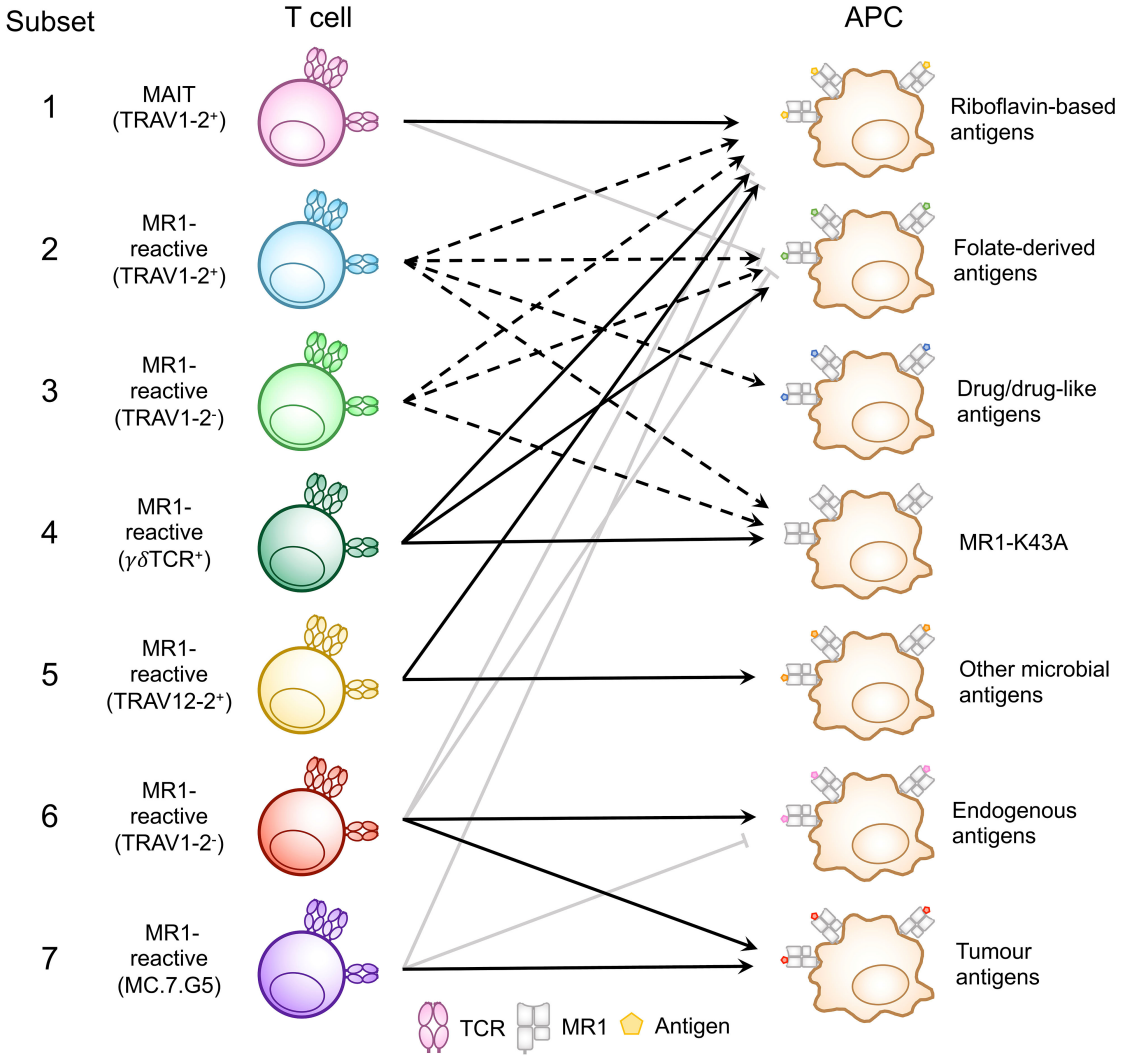
Classification of known MR1-reactive T cells based on MR1-antigen reactivity pattern. MR1-reactive T cells bear an αβ or γδ T cell receptor (TCR) that recognizes one or more classes of antigen presented by MR1 on antigen presenting cells (APCs). Solid point arrows indicate that all T cells within the subset recognize the specified antigen, broken point arrows indicate that some T cells within the subset recognize the specified antigen and gray block arrows indicate no recognition of the class of antigen. Table 1 outlines the frequency, TCR usage (where known) and surface characteristics of each subset using this classification. This schematic was inspired by Figure 1 from a recent commentary piece (91).

A range of MR1-antigen reactivity patterns have emerged for individual T cell clones and the TCRs they express. These reactivity patterns were identified based on (i) binding of T cell clones or TCR reporter cell lines to MR1-antigen tetramer (involving riboflavin-based antigens or other antigens) or mutated MR1 Lys43 to Ala (MR1-K43A) tetramers; and (ii) activation of T cell clones or TCR reporter cell lines in cellular assays with antigen presenting cells expressing physiological levels of MR1 or overexpressing MR1 or MR1-K43A. Notably, this is an active area of research and it is likely that additional reactivity patterns will emerge. Whilst the patterns observed so far are not always absolute, in that one reactivity might be less dominant, for simplicity they can be grouped as outlined below and illustrated in Figure 3 and Table 1. Similarly, a particular T cell phenotype (TCR usage, surface markers, transcription factors) can be exclusively or more frequently associated with one particular antigen reactivity pattern, although in some cases significant overlaps in TCR usage (and other phenotypic characteristics) have been observed for TCRs with different MR1-antigen reactivity patterns.

1. Reactivity to MR1-5-OP-RU/5-OE-RU (and less potent ribityllumazines) but not to other MR1-ligands. This reactivity is mediated by the population of cells referred to as MAIT cells, expressing the canonical MAIT TCR and phenotypic markers associated with the MAIT cell lineage in humans and mice (7, 24, 26, 28, 80). The same reactivity can also be mediated by a small fraction of human, TRAV1-2^−^ T cells with diverse TCR usage (e.g., clone MAV36 that expresses a TRAV36^+^ TCR), including some cells that feature a MAIT-like phenotype (CD161^+^, IL-18Rα^+^, CD218a^+^, CD26^+^) (80, 89). Notably, a follow-up study revealed that TRAV36^+^ MR1 reactive T cells are possibly a second public TCR family, alongside MAIT cells, capable of specifically recognizing riboflavin-based antigens in the context of MR1 (80, 89). The *TRAV36* gene segment of these TCRs was not rearranged with the classical MAIT *TRAJ* gene segments (*TRAJ33, TRAJ12, TRAJ20*), and hence lacked a Tyr95α (80, 89). Reminiscent of the classical MAIT TCRα chain, they displayed a largely germline-encoded CDR3α loop. In stark contrast to canonical MAIT TCRs, these TCRs featured nearly invariant CDR3β loops of constrained length (14 amino acids) (89). Structural studies on the MAV36 TCR revealed the CDR1a loop was predominant at the MR1 interface, including contacting the antigen directly, analogous to the role of the CDR3α loop of classical MAIT TCRs (80). Not surprisingly, among TRAV1-2^+^ T cells, MR1-5-OP-RU tetramer^+^ cells predominate, the majority of which express a classical MAIT phenotype, while amongst TRAV1-2^−^ cells, the frequency of MR1-5-OP-RU tetramer^+^ cells is similar to that of MR1-pterin tetramer^+^ cells [<0.2% of circulating T cells (80)], although it is perhaps higher among CD8^+^ T cells (88). Regardless of antigen recognition pattern, generally TRAV1-2^−^ MR1-reactive T cells are heterogeneous in surface markers and transcription factor profile, with the majority lacking CD45RO, PLZF, RORγt, and being heterogenous in T-bet expression, in line with developmental and functional differences compared to MAIT cells (80). Recently, novel MR1-reactive TCR rearrangements were identified from T cells in C57BL/6 mice deficient in the canonical MAIT junctional gene segment *TRAJ33* (89). In spite of genetic deletion of the *TRAJ33* gene segment, MAIT cells were detected using MR1-5-OP-RU tetramer, albeit at a dramatically reduced frequency (50-fold less) compared to wildtype mice (89). MR1-reactive T cells from *TRAJ33* knockout (KO) mice formed two distinct subsets based on TCR usage: those that expressed classical MAIT TCRs (TRAV1, Tyr95α) with more diverse junctional gene segments; most notably *TRAV1-TRA12/TRAJ9/TRAJ40* rearrangements, or MR1-reactive T cells with more diverse TCR usage altogether (non-TRAV1) (89). T cell clones that expressed the classical MAIT TCRs, derived from the *TRAJ33* KO mice, recognized MR1-5-OP-RU tetramers but not MR1-Ac-6-FP tetramers, verifying their specificity for the riboflavin-based antigens. In contrast, amongst the more diverse TRAV1^−^ MR1-reactive T cell clones, none were solely MR1-5-OP-RU specific (see recognition pattern 2) (89).
2. Reactivity to MR1-5-OP-RU/5-OE-RU (and less potent ribityllumazines) and other MR1-ligands. This reactivity involves ligand-dependent crossreactivity, a well-known concept in T cell biology. Examples include the cross-recognition of pterins (6-FP and Ac-6-FP) and pyrimidines (5-OP-RU) by <5% of human TRAV1-2^+^ T cells (e.g., clones AM1, AM2, AM3) that expressed a classical MAIT TCR, typically featuring the *TRAV1-2-TRAJ33* rearrangement and no discernible TCRβ motifs (80). Only a very small fraction of MR1-reactive TRAV1-2^−^ T cells (e.g., clone MAV14, TRAV14) in humans were identified to be crossreactive (80, 89). All of these TRAV1-2^−^ T cells expressed TCRs with diverse usage and only one TCR sequence featured the Tyr95α (80). Differential antigen recognition was a common feature among both TRAV1-2^+^ and TRAV1-2^−^ crossreactive T cells, whereby some TCRs preferred antigens of a specific class (riboflavin-based or folate-derived), while others were capable of distinguishing between antigens of the same class (6-FP or Ac-6-FP) (80). A TRAV1-2^−^ T cell clone (D462-E4, *TRAV12-2*) reacted to MR1-5-OP-RU tetramer and responded to RL-6-Me-7-OH but not RL-6,7-diMe and in addition to infection with *Streptococcus pyogenes* which lacks the riboflavin biosynthetic pathway, suggestive of an undefined riboflavin-independent MR1 antigen (88). The same clone also responded moderately to PLI, to a level similar as compared to RL-6-Me-7-OH (73). This clone did not express the MAIT cell marker CD161, although it might have been downregulated as a result of T cell expansion with anti-CD3 (88). The same research team also identified another TRAV1-2^+^ T cell clone (D481-C7) which also reacted with MR1-5-OP-RU tetramer, preferentially recognized RL-6,7-diMe over RL-6-Me-7-OH, and potently responded to PLI and PLIII (73). Some MAIT TCR expressing reporter cell lines and subsets of primary TRAV1-2^+^ MAIT cells also displayed crossreactivity to chemically diverse drugs, drug metabolites and drug-like molecules (29, 30).
3. Auto-reactivity to MR1, a more MR1-centric reactivity compared to antigen-centric reactivity. This type of reactivity was found amongst human TRAV1-2^+^ T cells, where clone M33-64, which expressed a classical MAIT TCR, stained with MR1 tetramer loaded with 5-OP-RU or pterin antigens as well as MR1-K43A tetramer. A matching TCR reporter cell line displayed MR1-dose dependent activation and limited antigen dose dependency (80). Surprisingly, a mutation of Tyr95α to Phe in the M33-64 TCR did not abolish MR1-5-OP-RU reactivity, in stark contrast to all previously studied MAIT TCRs, which was attributed to a dominant role of the CDR3β loop in both stabilizing the TCR and in making considerable contacts with MR1 (80). Autoreactivity in the context of MR1 overexpressing cell lines was also observed in <15% of mouse hybridomas derived from TAP^−/−^ C57BL/6 mice (clones 6C2, 6H2, 18G7, 8D12) (5, 6, 92–95) and <10% of hybridomas from Vα19Tg Cα^−/−^ mice, all featuring canonical MAIT TCRs (94). Interestingly, based on extensive mutagenesis, the 6C2 hybridoma used overlapping but distinct TCR residues for the recognition of MR1 on *E. coli* infected cells vs. MR1 overexpressing cells in the absence of infection (95). Recently MR1-autoreactivity was also found to be mediated by γδ TCR^+^ T cells, present in human PBMCs (<0.001 to 0.1% of CD3^+^ T cells and from <0.1 to 5% of γδ T cells) and in tissues (liver, stomach, lung, and duodenum), the frequencies of which may be enriched in association with some diseases (96). Some MR1-reactive γδ TCRs bound underneath the antigen binding cleft (contacting primarily the α3 domain of MR1), while others interacted with the antigen binding cleft of MR1, akin to the classical MAIT αβ TCR. The former type was observed in blood of 6 out of 20 individuals. Activation of γδ TCR expressing reporter cell lines varied and was dependent on MR1 with some potential for modulation by MR1-bound antigen. MR1-reactive γδ TCRs were diverse in gene usage. Most (72%) used TRDV1; the remainders, aside from one TRDV5^+^ clone, expressed TRDV3. Whilst all functional TRGV genes including TRGV2, 3, 4, 5, 8, and 9 were found amongst MR1-reactive γδ T cells, TRDV1^+^ and TRDV3^+^ TCRs predominantly paired with TRGV8. MR1-5-OP-RU tetramer^+^ γδ T cells were mostly CD4^−^CD8α^−^ or CD8α^+^ with variable CD161 expression. Thus, they resembled other cells of the γδ T cell lineage and appeared phenotypically diverse (96). Whether MR1-reactive γδ T cells exist in mice or other species remains to be investigated.
4. Reactivity to other MR1-ligands but not MR1-5-OP-RU/5-OE-RU (and less potent ribityllumazines). Such reactivity pattern was first observed for the TRAV1-2^−^ MAV21 TCR (TRAV21) which specifically recognized pterins but not 5-OP-RU (80). Recently, Crowther et al. identified another TRAV1-2^−^ T cell clone in blood of a healthy individual (MC.7.G5, *TRAV38.2/DV8-TRAJ31, TRBV25.1 TRBJ2.3*) which specifically recognized an antigen/s that was expressed or upregulated in cancerous cells but not non-cancerous cells (resting, activated, stressed or infected), in an MR1-dependent manner (90). Interestingly, the observed T cell reactivity was donor-unrestricted, with multiple HLA-mismatched tumor cells recognized in an MR1-dependent manner, whilst not exerting allo-reactivity, making this T cell clone particularly interesting for immunotherapies (90). Potent activation, including cytotoxicity, was achieved with physiological levels of MR1 and high effector (T cell) to target (APC) ratios. Both Ac-6-FP and bacterial riboflavin-based antigens were not recognized and acted as competitive inhibitors of activation. Whilst a clone with a similar reactivity pattern was also isolated from a second donor, the frequency of similar clones within an individual and their prevalence in the general population and in the context of cancers are unknown. Also, the phenotype of the clones (other than the TCR of one of them) was not described which may provide insights into its developmental origin. Previously, Lepore et al. (44) had identified a population of T cells in healthy individuals that comprised 1 in 2,500–5,000 of circulating T cells. Designated “MR1T” cells, clones of these cells recognized, in the context of physiological levels of MR1 and dependent on MR1, endogenous cellular antigens from various tissues, including cancer tissues, and thus were not cancer specific. One MR1T cell clone was TRAV1-2^+^ (clone DGA4), but most were TRAV1-2^−^, expressing diverse TCRα and β chains (e.g., clone DGB129; *TRAV29/DV5-TRAJ23*; and *TRBV12-4-TRBJ1-1*) (44). Like the MC.7.G5 clone, MR1T cell clones (e.g., DGB129) did not recognize 6-FP and their activation was inhibited by 6-FP. However, unlike the MC.7.G5 clone, which did not bind MR1-K43A tetramer or respond to MR1-K43A overexpressing cells (90), MR1T cell clones equally recognized wild type and MR1-K43A overexpressing cells. This suggested to the authors that relevant antigens that were recognized did not depend on Schiff base formation with MR1 for TCR recognition but may also suggest an MR1-centric recognition or MR1 autoreactivity by some MR1T cells for which 5-OP-RU/ribityllumazine are permissive antigens but pterins are not. At least two clones (DGB129 and DGB70) did however, differentially recognize fractions of cell/tumor lysates, demonstrating differential antigen specificity for at least some clones. With the exception for one MR1T clone, all TRAV1-2^−^ clones were CD161^−^ and all, except for one, were CD8α^+^ (DGB129, TRAV1-2^−^, CD161^−^, CD8α^+^; DGB70, TRAV1-2^−^ [TRAV5], CD161^−^, CD8^−^). DGA4, the TRAV1-2^+^ clone, was CD161^+^, CD8^−^. Phenotypic and functional characterization of MR1T cell clones showed multiple chemokine receptor expression profiles and secretion of diverse effector molecules, suggesting functional heterogeneity within this population that was also distinct from that of MAIT cells. In a T-helper like function, clone DGB129 induced maturation of monocyte derived dendritic cells and another clone suggested the possible contribution of MR1T cells to intestinal epithelial barrier homeostasis.

**Table 1:** Frequency, TCR usage (where known) and surface phenotype of each subset of MR1-reactive T cells as depicted in Figure 3.

**Subset**		**Reactivity pattern**	**Frequency**	**TCR usage**	**Surface phenotype**	**References**
1	MAIT (TRAV1-2^+^)	1	Human: 3% of blood T cells Mouse: 0.1% of blood T cells, 0.1–5% in peripheral organs	Human: TCRα: TRAV1-2-TRAJ33, -TRAJ12, -TRAJ 20 TCRβ: TRBV6-1, TRBV6-4, TRBV20 Mouse: TCRα: TRAV1-TRAJ33, -TRA12, -TRAJ9, -TRAJ40 TCRβ: TRBV19, TRBV13	Human: TRAV1-2, CD161, ±CD4, ±CD8, IL-18R, CD26, ±CD56, CD45RO, CD218a, CD127 Mouse: ±CD4, ±CD8, ±NK1.1, CD44, ±CD103, CD127, CXCR6, IL-18R	(5, 7, 8, 10, 11, 24, 36, 38, 89)
2	MR1-reactive (TRAV1-2^+^)	1, 2, 3	Human: <0.2% of blood T cells Mouse: ND	Human: TCRα: TRAV1-2-TRAJ33 TCRβ: TRBV6-2, TRBV6-4, TRBV20, TRBV4, TRBV5 Mouse: TCRα: TRAV1-TRAJ9 TCRβ: TRBV13-3	Human: TRAV1-2, ±CD161, ±CD45RO, CD8, ±CD56 Mouse: CD44	(29, 30, 80)
3	MR1-reactive (TRAV1-2^−^)	1, 2, 3	Human: <0.2% of blood T cells Mouse: ND	Human: TCRα: TRAV36-TRAJ34 (lack Tyr95α), diverse TCRβ: TRBV28-TRBV2-5, diverse Mouse: TCRα: TRAV16, diverse TCRβ: TRBV13, diverse	Human: ±CD161, ±CD45RO, CD8, ±CD218a ±CD26 Mouse: CD44	(80, 89)
4	MR1-reactive (gd TCR^+^)	3	Human: <0.1% of blood T cells	Human: TCRβ: TRDV1, TRDV3, TRDV5 TCRβ: TRGV8, TRGV5, TRGV4, TRGV3, TRGV2, TRGV9	Human: ±CD161, ±CD4, ±CD8	(96)
5	MR1-reactive (TRAV12-2^+^)	2	Human: ND	Human: TCRα: TRAV12-2-TRAJ39 TCRβ: TRBV29-1, TRBJ1-5	Human: CD8, CD45, CD26, CD161^−^	(73, 88)
6	MR1-reactive (TRAV1-2^−^)	4	Human: <0.04% of blood T cells	Human: TCRα: diverse TCRβ: diverse	Human: ±CD8, ±CD161	(44)
7	MR1-reactive (MC.7.G5)	4	Human: ND	Human: TCRα: TRAV38-2/DV8-TRAJ31 TCRβ: TRBV25-1-TRBJ2-3	Human: CD8	(90)

*ND, not determined*.

## Discussion

We are beginning to appreciate the diversity of microbial and endogenous (including cancer) antigens presented by MR1 and the heterogenous populations of T cells which recognize them. Independent and repeated identification of these cells has led to the classification “MR1-reactive T cells,” illustrated in Figure 3 and Table 1, inclusive of MR1-restricted MAIT cells, and more phenotypically and functionally heterogenous T cells, some of which are MAIT-like in their phenotype, grouped together as “other MR1-reactive T cells” (44, 80, 89, 91). Whilst TRAV1-2^+^ T cells are dominant amongst MAIT cells, other MR1-reactive T cells mostly include TRAV1-2^−^ αβ TCR^+^ T cells (44, 73, 80, 89, 90) as well as γδ TCR^+^ T cells (96). Thus, the current stage of research suggests that most MR1-reactive T cells, namely MAIT cells, feature a biased TCR α-chain, and react with a limited repertoire of pyrimidine and ribityllumazine antigens derived from riboflavin biosynthesis. There are also several examples of TCR β-chain usage influencing antigen preference and allowing for antigen crossreactivity by classical MAIT TCRs, and future work might identify additional examples of physiological relevance. The emerging descriptions of other MR1-reactive T cells highlight that the classical MAIT TCR α-chain is not the only solution that warrants MR1-reactivity, however, more work is needed to validate and corroborate the TCR usage and antigen specificities of other, more diverse T cells. In particular, determining the chemical identities and biosynthetic origins of the tumor-associated and endogenous antigens recognized by some of the other MR1-reactive T cells will allow the generation of tetramers which will greatly assist in determining the prevalence of these cells as well as their potentially important role in anti-tumor immunity and other immune-functions. It will also help to better characterize the phenotype (including the TCR usage) and of this diverse set of T cells including public as compared to private existence of individual clones, recently described for TRAV36^+^ MR1-reactive T cells (89). Another interesting question concerns the lineage development of other MR1-reactive T cells that feature a non-MAIT phenotype. Is it possible that these cells might have originated from another T cell lineage and cross-react across MHC families, including classical MHC molecules (MHC-I and MHC-II) and MHC-I-like molecules? In TCR α-chain transgenic mice that lack MR1 (*V*α*19iC*α^−/−^*MR1*^−/−^) MR1-5-OP-RU tetramer^+^ T cells make up ~32% of T cells in the spleen as compared to ~50% in mice proficient in MR1 (*V*α*19iC*α^−/−^) (97). The population of these cells mediated both MR1-dependent and MHC-I (H2-K^b^/H2-D^b^) dependent reactivity *in vitro* (97), although it was not investigated whether these specificities were mediated by single T cells and single TCRs. More recently, MR1-5-OP-RU tetramer^+^ T cells featuring the canonical MAIT TCRα chain (*TRAV1-TRAJ33*) but not the typical TRBV13 bias were also found in very low percentages in the thymus of non-transgenic mice, *Mus musculus castaneus* (CAST mice), modified to lack MR1 (86). Thus, MR1-5-OP-RU tetramer^+^ T cells can develop in the absence of MR1, inferring selection may occur on other MHC molecules. Indeed, MR1 is highly conserved with HLA-A2 (39% sequence conservation), suggesting mimicry as a potential mode of crossreactivity by these cells. So far evidence of peripheral T cells that crossreact between classical MHC molecules and MHC-I-like molecules remains elusive.

## Author Contributions

MS and SE wrote and revised the manuscript, read, and approved the submitted version. All authors contributed to the article and approved the submitted version.

## Conflict of Interest

SE is an inventor on patents describing MR1 antigens and MR1 tetramers. The remaining author declares that the research was conducted in the absence of any commercial or financial relationships that could be construed as a potential conflict of interest.
